# Evaluating the Usability, Acceptability, User Experience, and Design of an Interactive Responsive Platform to Improve Perinatal Nurses’ Stigmatizing Attitudes Toward Substance Use in Pregnancy: Mixed Methods Study

**DOI:** 10.2196/67685

**Published:** 2025-05-08

**Authors:** Michael Rubyan, Yana Gouseinov, Mikayla Morgan, Deborah Rubyan, Divya Jahagirdar, David Choberka, Carol J Boyd, Clayton Shuman

**Affiliations:** 1 Department of Health Management and Policy School of Public Health University of Michigan Ann Arbor, MI United States; 2 Institute for Healthcare Policy and Innovation University of Michigan Ann Arbor, MI United States; 3 Department of Systems, Populations, and Leadership School of Nursing University of Michigan Ann Arbor, MI United States; 4 Honey Locust Health West Bloomfield, MI United States; 5 Museum of Art University of Michigan Ann Arbor, MI United States; 6 Center for the Study of Drugs, Alcohol, Smoking and Health University of Michigan Ann Arbor, MI United States; 7 Women’s and Gender Studies University of Michigan Ann Arbor, MI United States

**Keywords:** digital platform, nurse training, usability, user experience, user interface, mixed methods, theatre testing, stigma, perinatal substance use, art intervention, acceptability, perinatal, substance use, pregnancy, perinatal nurse, feasibility, interactive responsive platform

## Abstract

**Background:**

Perinatal nurses are increasingly encountering patients who have engaged in perinatal substance use (PSU). Despite growing evidence demonstrating the need to reduce nurses’ stigmatizing attitudes toward PSU, limited interventions are available to target these attitudes and support behavior change, especially those reflecting the overwhelming evidence that education alone is insufficient to change practice behavior. Arts-based interventions are associated with increasing nursing empathy, changing patient attitudes, improving reflective practice, and decreasing stigma. We adapted ArtSpective for PSU—a previously evaluated, in-person, arts-based intervention to reduce stigma toward PSU among perinatal nurses—into an interactive, digital, and responsive platform that facilitates intervention delivery asynchronously.

**Objective:**

This study aimed to evaluate the usability, acceptability, and feasibility of the interactive, responsive platform version of ArtSpective for PSU. Our goal was to elicit the strengths and weaknesses of the responsive platform by evaluating the user experience to identify strategies to overcome them.

**Methods:**

This study used a mixed methods approach to explore the platform’s usability, user experience, and acceptability as an intervention to address stigma and implicit bias related to PSU. Theatre testing was used to qualitatively assess usability and acceptability perspectives with nurses and experts; a modified version of the previously validated 8-item Abbreviated Acceptability Rating Profile was used for quantitative assessment. Quantitative data for acceptability and satisfaction were analyzed using descriptive statistics. All qualitative data were analyzed iteratively using an inductive framework analysis approach.

**Results:**

Overall, 21 nurses and 4 experts in stigma, implicit bias, and instructional design completed theatre-testing sessions. The mean duration of interviews was 31.92 (SD 11.32) minutes for nurses and 40.73 (SD 8.57) minutes for experts. All participants indicated that they found the digital adaptation of the intervention to be highly acceptable, with mean acceptability items ranging from 5.0 (SD 1.0) to 5.5 (SD 0.6) on a 1-6 agreement scale. Nurses reported high satisfaction with the platform, with mean satisfaction items ranging from 5.14 (SD 0.56) to 5.29 (SD 0.63) on a 1-6 agreement scale. In total, 1797 interview segments were coded from the theatre-testing sessions with 4 major themes: appearance, navigation, characterization, and overall platform, and 16 subthemes were identified. Consistent with the quantitative findings, the results were positive overall, with participants expressing high satisfaction related to the platform’s appearance, the ease with which they could navigate the various modules, engagement, clarity of the presentation, and feasibility of being completed asynchronously.

**Conclusions:**

Developing and evaluating the usability of a digital adaptation of ArtSpective for PSU resulted in strong support for the usability, acceptability, and satisfaction of the program. It also provided insight into key aspects related to acceptability and usability that should be considered when designing a digital adaptation of an arts-based intervention for health care providers.

## Introduction

### Background

Stigmatizing attitudes among nurses who treat patients with perinatal substance use (PSU) [1-7] is a major barrier to the implementation of evidence-based practices for maternally provided care (ie, breastfeeding and skin-to-skin contact) that improve infant outcomes; decrease symptom severity; and reduce pharmacologic treatment, length of stay, and costs [8-18]. Despite growing evidence that demonstrates the need to reduce nurses’ stigmatizing attitudes toward PSU [17-20], limited interventions are available that target these attitudes and support behavior change among nurses. Interventions that have been studied use didactics to increase awareness about the need to reduce stigma and increase compassion [20]. However, these interventions do not reflect the overwhelming evidence that education alone is not sufficient to change practice behavior [21,22] and that stigmatized attitudes may prevent the application of education to individual practice [23]. Evidence-based interventions targeting stigmatizing attitudinal change are necessary to empower nurses to effectively reduce biases and judgments toward PSU and care for these patients.

One of the key weaknesses of traditional interventions in addressing stigma is that they are generally structured around passive learning and focus on knowledge deficits rather than intervening on stigma itself [24]. In contrast, increasing research demonstrates how arts-based interventions improve clinical skills and communication between health care providers, especially for stigmatized attitudes [25]. Arts-based pedagogy is associated with increasing nursing empathy, changing stigmatized attitudes toward patients, and improving reflective practice [26]. Photography and digital storytelling have been demonstrated to decrease stigma and increase provider willingness to help patients affected by substance use disorder [27]. In addition, creative storytelling has been effectively used to improve clinician attitudes toward persons with dementia, a highly stigmatized population [28].

### ArtSpective for PSU: An Arts-Based Intervention

#### Overview

Based on existing evidence of efficacy for arts-based interventions to improve clinician attitudes toward stigmatized patient populations [29,30], we developed ArtSpective for PSU to facilitate perspective taking and change attitudes toward PSU among nurses to facilitate their engagement with mothers and promote evidence-based practice that is not impeded by biases and stigma. ArtSpective for PSU was initially designed as a synchronous intervention that is delivered in one 60-minute session to a small group of nurses through live videoconference [24,31-33]. The intervention is grounded in art pedagogy previously used by art museum educators and facilitates a perspective-taking exercise using curated fine-art photographs and guided narrative storytelling. Participants are shown images and instructed to select two to interact with for the intervention. Selected images use a documentary photographic style characterized by ambiguity in subject matter, which enables participants to write two short creative narratives from two different perspectives (one perspective per image): (1) the perspective of a subject in the photograph and (2) the outside perspective of the nurse. Perspective taking is described as taking the point of view of another person within the other's context. This is grounded in evidence that this type of contextualization is associated with taking actions and exerting behaviors to alleviate distress and improve outcomes [34]. Participants spend time discussing their narratives with partners during the synchronous session after writing a narrative about the photographs they reviewed. They also engage in facilitated dialogue related to the perspective-taking attitudes elicited during their participation. These conversations enabled participants to compare their responses to the photographs and notice when their interpretations displayed biased attitudes.

While participants previously reported high demand, satisfaction, and efficacy with the synchronous version of ArtSpective for PSU [24], they identified key barriers to scaling the intervention across hospitals and health systems, given its synchronous and in-person modality. These barriers are consistent with several implementation barriers that prevent engagement with arts-based interventions, including fluctuation in participation, skepticism of efficacy, and obstacles related to delivery, especially in person [35]. Given these barriers, we adapted ArtSpective for PSU into an interactive, responsive platform that facilitates the delivery of the intervention asynchronously. Responsive platforms and digital delivery of interventions have been demonstrated to improve uptake and overcome implementation barriers related to scalability, especially for interventions designed for nurses [36-42].

#### Design and Development of Digital Intervention

**Figure 1:**
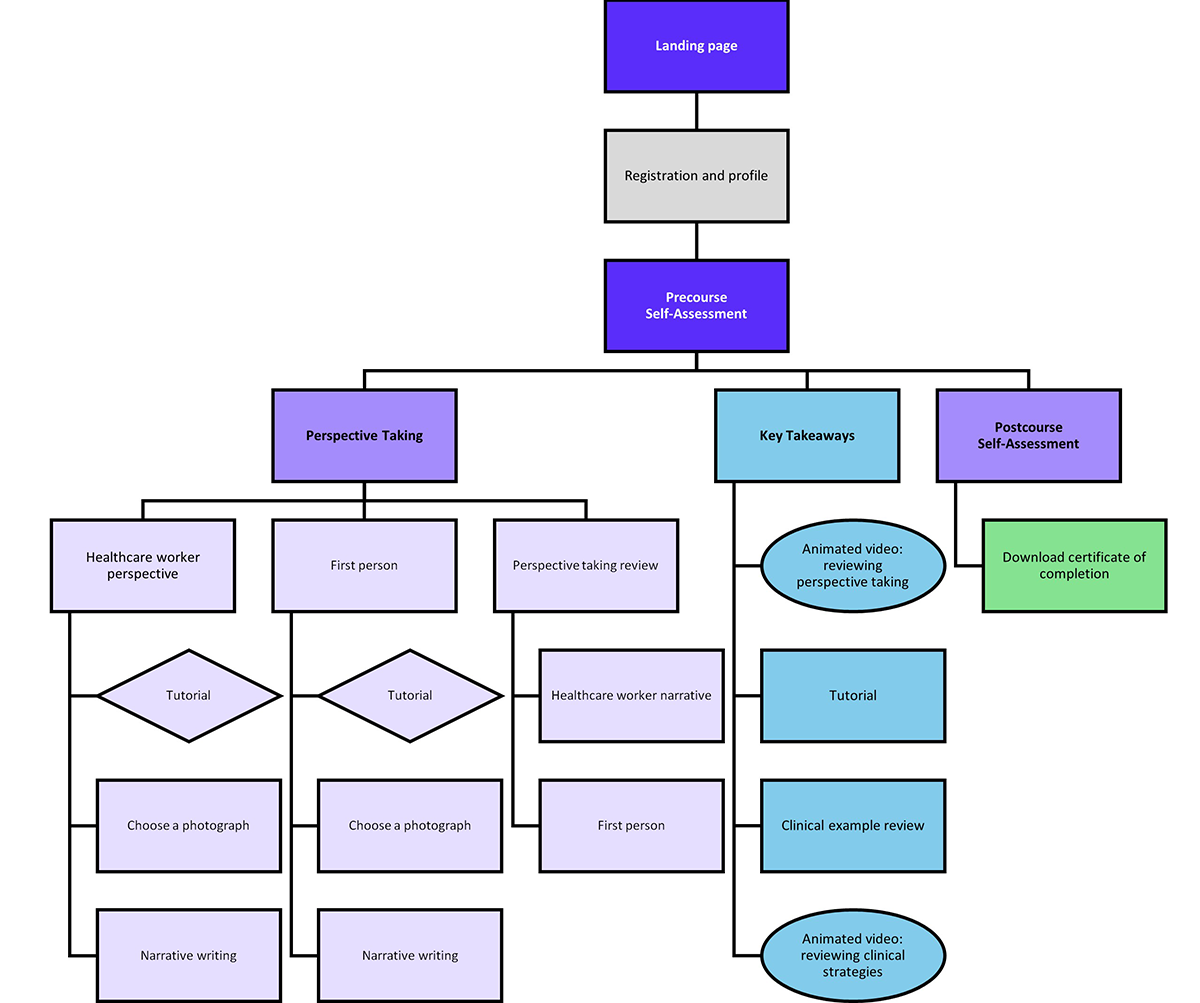
ArtSpective for perinatal substance use (PSU) platform structure.

A multidisciplinary team, including experts in nursing, labor and delivery, health informatics, health communication, and implementation science, developed the interactive responsive platform. Before developing the platform, we conducted 2 focus groups related to the synchronous version of the intervention (n=11) to identify specific requirements for the interactive, responsive platform. Participants noted the need for the adapted intervention to (1) take less time to complete, (2) allow for self-pacing, and (3) be delivered asynchronously [24]. Partnering with a public health communications firm, the synchronous intervention components were adapted into an interactive, responsive platform optimized for desktop, smartphone, or tablet interface. The platform incorporated electronic art exhibits and facilitation materials consisting of a 30-minute, web-based session that nurses could complete asynchronously (Figure 1). The platform design is grounded in the user experience design methodology, which is anchored in principles related to understanding users, user participation throughout the process of development, user-centered evaluation, iteration, a reflection of the entire user experience, and a multidisciplinary perspective to design and development [43,44]. The overall design is guided by Material 3, an iteration of Google Material Design, an open-source adaptable system of guidelines, components, and tools that support best practices of user interface design [45]. The platform also uses structured headings, alternative text descriptions, bold fonts, high-contrast color combinations, and captions to ensure accessibility across all users.

Before the study’s launch, the research team and health communications firm developed an interactive prototype of the digital adaptation of ArtSpective for PSU in Figma (Adobe). A panel of experts with extensive experience in perinatal nursing, PSU, art pedagogy, implementation science, and user interface and experience design examined all prototype elements in an iterative process as part of a heuristic evaluation. This process consisted of identifying platform functionalities individually based on the list of heuristics for usable interfaces [46,47]. The health communications firm iteratively refined the prototype as user interface issues were identified.

#### Platform Flow

**Figure 2:**
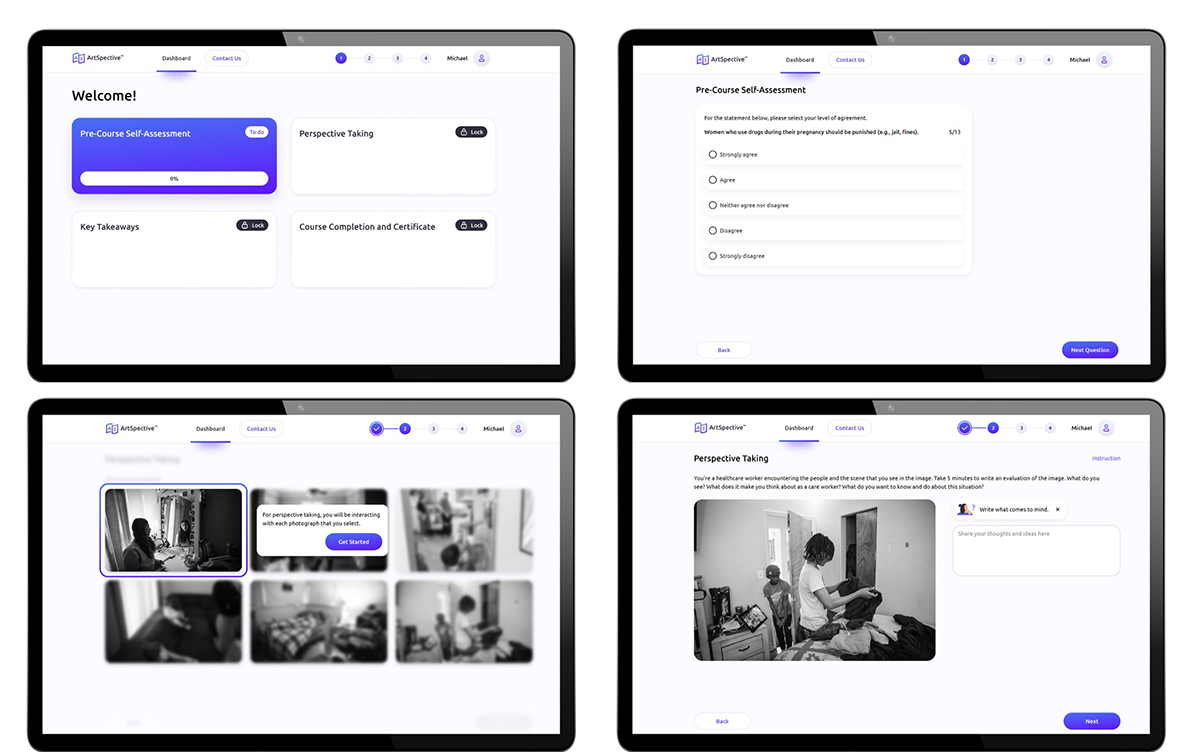
Sample of the dashboard, along with the Pre-Course Self-Assessment and Perspective Taking modules, in the adapted intervention.

At the platform launch, users are asked to register for an account or log into their existing account. Following account creation, users complete a series of demographic questions as part of creating their profile. Once a profile has been completed, the user lands on a dashboard with the four core modules that comprise the intervention: (1) *Pre-Course Self-Assessment*, (2) *Perspective Taking*, (3) *Key Takeaways*, and (4) *Course Completion and Certificate*. Modules are displayed as symmetrical cards with large rounding on each card. Small, rounded badges are included on each module card to indicate if it has a status of “To Do,” “Locked,” or “Completed.” Navigation is contained at the top of the interface, along with a module progress bar that uses numbers and colors to indicate progression through the platform (Figure 2).

In the Perspective Taking module (Figure 2), participants are presented with 6 rounded symmetrical cards containing photographs to select from for the exercise. Before choosing a photograph, the user is prompted to complete a brief guided tutorial and then prompted to imagine that they are a health care worker encountering the scene they see in the image and directed to type a message into a textbox consisting of an evaluation of the image from their health care worker perspective. Following this, the participant is presented with the same 6 photographs (the first photograph they chose has a reduced opacity to indicate that it can no longer be selected). The participant is prompted to type a message into a different textbox that evaluates the second image from their personal perspective. After entering their messages, the participant sees each photograph they chose and can read the messages they typed without editing them.

**Figure 3:**
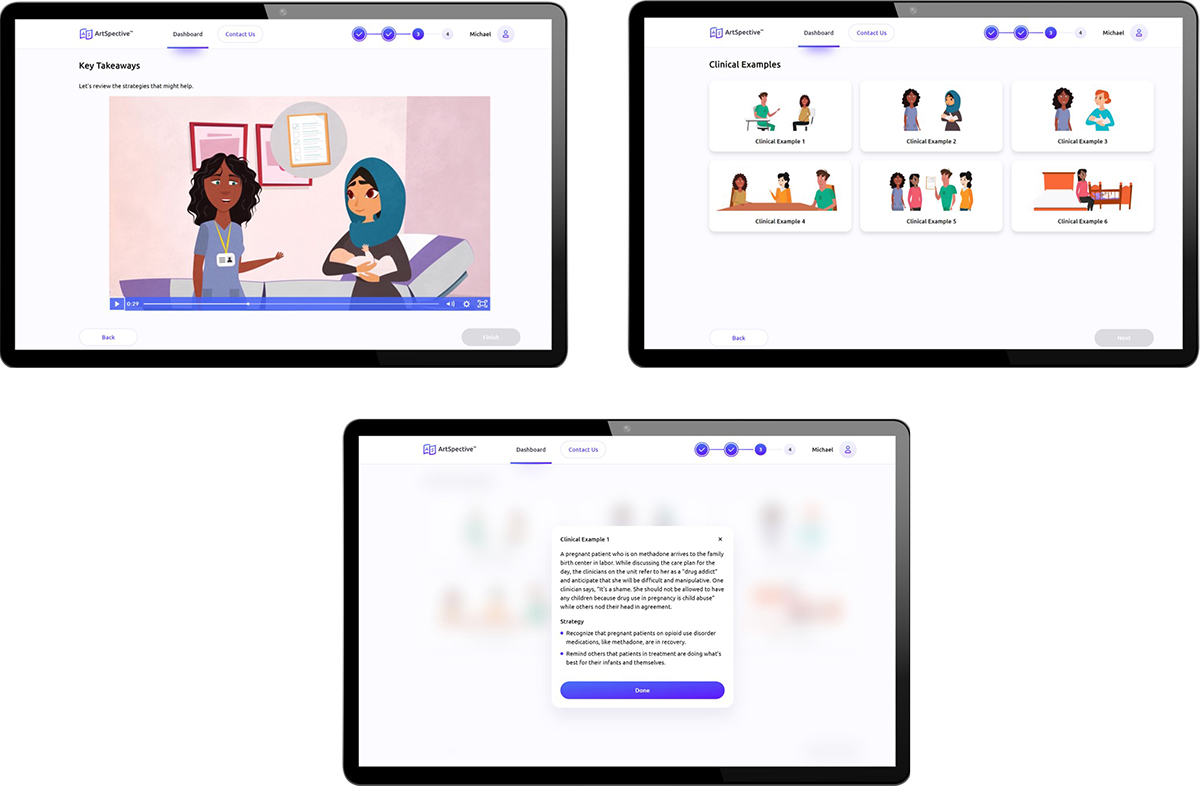
Sample of the video displayed in the Key Takeaways module and the Clinical Examples page presented in the adapted intervention.

In the Key Takeaways module (Figure 3), participants watch a 1-minute animated video depicting the perspective flipping exercise through the actions of animated characters, exploring how to limit assumptions in patient care through the lens of how the participant’s health-care-worker perspective may have been different from their personal perspective in the exercise. After watching the video, participants review 6 rounded symmetrical cards that contain clinical examples and strategies illustrated with the characters from the animated video and watch a 90-second video articulating the core clinical examples and techniques that users reviewed in the previous phase to reinforce the content. On average, participants spend 10 minutes engaging with this module.

Before evaluating the effectiveness of a digital adaptation, it is critical to examine its use and sustainability [48,49], including usability, acceptability, and feasibility with end users. Usability is characterized by how well specific users achieve a predetermined goal with efficiency and satisfaction in a particular context [50]. Acceptability relates to how end users think and feel about a digital intervention, while feasibility relates to how well it can be deployed with end users [51-53].

### Purpose

This study aimed to evaluate the usability, acceptability, and feasibility of the interactive, responsive platform version of ArtSpective for PSU—designed to deliver an adapted version of the in-person, arts-based, perspective-taking intervention to improve nurses’ stigmatizing attitudes toward PSU—following a mixed methods approach. Our goal was to elicit the strengths and weaknesses of the responsive platform by evaluating the user experience to identify strategies that overcome the weaknesses.

## Methods

### Study Design

The evaluation of the platform’s usability, acceptability, and feasibility followed a mixed methods approach, combining target user experience evaluation facilitated through Zoom (Zoom Communications) videoconference and a validated measure of acceptability.

### Procedure

#### Study Population and Recruitment

A purposive sample of nurses was invited to participate through an email sent to all nurses meeting eligibility criteria employed by a large, urban academic medical center in Chicago with high rates of birthing individuals with opioid use disorder and infants with neonatal opioid withdrawal syndrome. This was followed by snowball sampling in both community hospitals and academic health centers in Michigan and Chicago. Inclusion criteria consisted of (1) being credentialed as a registered nurse, (2) actively practicing in a perinatal unit (labor, postpartum, and neonatal intensive care unit), and (3) being at least 18 years old. In addition, a convenience sample of experts in instructional design, stigma, and implicit bias was recruited to participate. The target sample size was 24 nurses and 4 experts.

#### Methodology

We used theatre testing to qualitatively explore nurses’ and experts’ usability and acceptability perspectives. Theatre testing is a methodology that is integrated into the Assessment Decision Administration Production Topical Experts-Integration Training Testing (ADAPT-ITT) model and designed to facilitate the adaptation of evidence-based interventions [54]. It is a type of pretesting methodology that has been previously used to test messaging, such as television or print advertisements, with a key target demographic [55]. Participants who reflect the intended audience of the intervention are asked to respond to a demonstration of an intervention and answer questions designed to elicit their reactions to it [54]. A key strength of this methodology is its ability to rapidly gather feedback about a prototype that reflects the intervention as end users would experience it. We modified the 8-item Abbreviated Acceptability Rating Profile (AARP) [56], a previously validated and widely used acceptability instrument, for quantitative acceptability assessment. The abbreviated nature of the instrument was designed to overcome barriers related to time-intensiveness. Modifications to the AARP for this study included specific references to perinatal substance. We complemented the instrument with a separate stand-alone set of 5 items related to satisfaction that were used previously when conducting concept testing of the synchronous intervention [24].

Theatre testing for qualitative evaluation provides a deeper understanding of users’ perspectives on interface and platform design and individual user experience. Simultaneously, the quantitative data derived from the validated scale and our supplemental satisfaction questions provide precise measurements of acceptability and feasibility among nurses and experts. The goal was to explore both the usability and user experience of the platform and its acceptability and feasibility as an intervention to address stigma and implicit bias related to the treatment of PSU.

### Data Collection

A total of 2 research team members (MR and CS) designed the theatre-testing session interview guide, which was piloted for presentation and clarity of information and workflow (Multimedia Appendix 1). MR (male) has a PhD and MPH. He is a clinical assistant professor with experience and training in implementation science and provider behavior change. CS (male) has a PhD, MSN, and RN. He is an assistant professor with training and experience in implementation science and care for vulnerable populations, including individuals with PSU and postpartum women. This study is reported according to the COREQ (Consolidated Criteria for Reporting Qualitative Research) guidelines (Multimedia Appendix 2) [57]. Participants were invited by email to individual sessions facilitated through Zoom videoconference and were informed that the researchers leading the study were developing a new platform to promote a behavior change intervention. Sessions lasted between 30 and 45 minutes between December 2022 and October 2023. Each session was conducted by one of two analysts on the research team (YG) who have extensive expertise in qualitative interviewing and have clinical experience caring for patients with substance use disorder. The analysts did not share their assumptions or motivations for facilitating the study with participants. The study procedure was explained to participants after they had logged into the Zoom session, and participants were informed that the interview would be video recorded and transcribed. Interviewers used the screen-sharing feature in Zoom to display the interactive prototype and clicked through the screens and prompts. To understand participant reactions to the platform, the concurrent think-aloud method was used by asking participants to verbalize what they noticed or did not understand [58]. No training or contextualization of the prototype were offered before the interview began to avoid biases related to preparation. Participants were given minimal assistance in understanding what was displayed, and interviewers only interjected with contextual information to encourage them to continue reflecting on what they saw. Following the demonstration, the interviewer debriefed each participant, using a semistructured interview guide that assessed overall user experience, design of the content in the platform, and ease of navigation, and performed a brief retrospective analysis of issues that participants noted during the demonstration [59]. Participants also completed a questionnaire using Qualtrics XM hosted at the University of Michigan that included questions about demographics, the AARP, and stand-alone satisfaction questions.

Nonparticipants did not attend the sessions, and no relationship with participants was established before they engaged with the study. No repeated interviews were conducted, and no field notes were taken. Sessions were recorded through Zoom Recordings, pseudonymized, and transcribed verbatim. Transcripts were not returned to participants for their review. Data were transcribed by Rev. Data were analyzed in MAXQDA (version 22.8.0; VERBI GmbH), a computer-assisted qualitative data analysis software. The research team met after 21 interviews with nurses to discuss data saturation and information power. Since no new themes emerged in later interviews, the research team concluded that information power had been reached, and no additional interviews were required. In addition, following the 4 interviews with experts, the research team met separately to discuss information power and concluded that since all 4 experts had provided similar feedback, information power had been reached.

### Data Analysis

**Figure 4:**
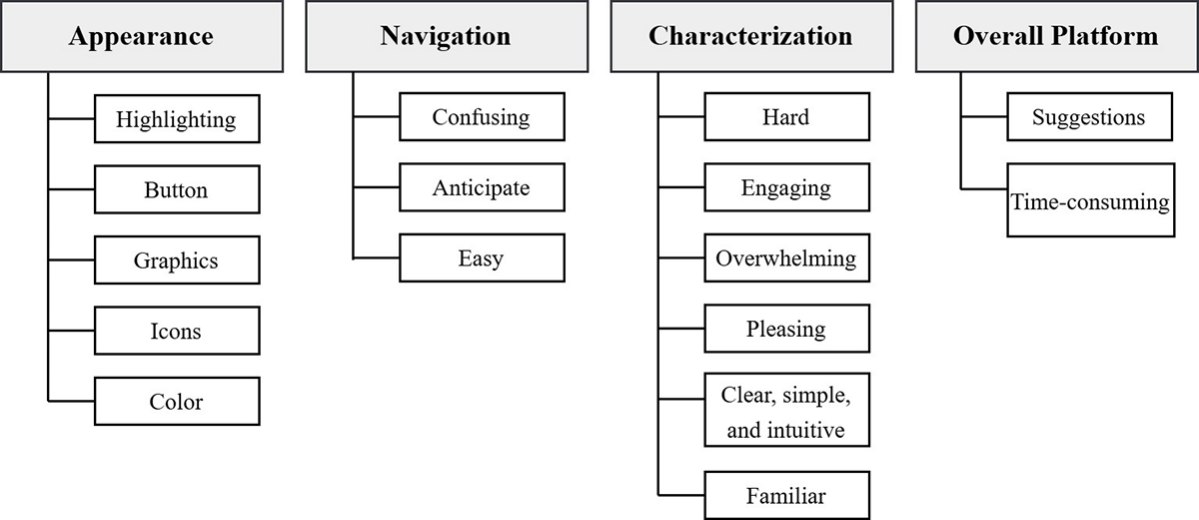
Overview of overall themes and subthemes.

All data, including pilot sessions, were included in the final data analysis. Pilot sessions were included, as no changes were made to the interview guide or protocol after the pilot sessions were conducted. We conducted separate quantitative and qualitative data analyses. All quantitative data regarding acceptability and satisfaction were checked for completeness, and analyses were performed using descriptive statistics (mean, SD, frequency, and percentage) to describe demographic characteristics, responses to the modified AARP, and satisfaction items. All qualitative data were analyzed iteratively, using an inductive framework analysis approach [60]. Three multidisciplinary research team members with backgrounds in implementation science, perinatal nursing, and qualitative research methods (MR, YG, and MM) read the transcripts to inductively identify initial sets of codes. The team then met to discuss codes and map them to a coding schema, resulting in a codebook based on overarching themes. In addition, 2 research team members (YG and MM) independently coded segments in each transcript, meeting regularly with the entire research team to discuss discrepancies and refine or modify the codebook as necessary. This resulted in an iteratively developed, more detailed codebook. All themes were organized into major themes and subthemes that reflected quotes from the interview transcripts (Figure 4). Participants did not provide feedback on the findings.

### Ethical Considerations

The study was deemed exempt by the University of Michigan institutional review board (HUM00223937). Informed consent was obtained from all participants. Data have been deidentified. Participants received a US $30 cash card for their time.

## Results

### Overview

A total of 21 nurses and 4 experts completed theatre-testing sessions. No participants refused to participate. The mean duration of interviews was 31.92 (SD 11.32) minutes for nurses and 40.73 (SD 8.57) minutes for experts. Table 1 shows the demographic data of the participants. The majority of participants identified as female (22/25, 88%), and nearly half (12/25, 48%) of the participants were between 18 and 34 years old. Nearly half (10/21, 48%) of nurses practiced in the neonatal intensive care unit. Years of experience among nurses were equally divided across the three categories (<5, 5-10, and >10 years).

**Table 1:** Demographic characteristics of study participants (N=25).

Demographic characteristics		Value, n (%)
**Sex**		
	Male	2 (8)
	Female	22 (88)
	Other	1 (4)
**Race**		
	White	16 (64)
	African American	3 (12)
	Asian	4 (16)
	Other	2 (8)
**Ethnicity**		
	Hispanic	2 (8)
	Not Hispanic	23 (92)
**Age category (years)**		
	18-34	12 (48)
	>35	13 (52)
**Unit type (nurses only; n=21)**		
	NICU^a^	10 (48)
	Labor and delivery	6 (28)
	Postpartum	5 (24)
**Years in practice (nurses only; n=21)**		
	<5	7 (33)
	5-10	7 (33)
	>10	7 (33)

^a^NICU: neonatal intensive care unit.

### Quantitative Measures

All participants indicated that they found the digital adaptation of the intervention to be highly acceptable, with mean acceptability items ranging from 5.0 (SD 1.0) to 5.5 (SD 0.6) on a 1-6 agreement scale (Table 2). Nurses reported high satisfaction with the platform, with mean satisfaction items ranging from 5.14 (SD 0.56) to 5.29 (SD 0.63) on the same agreement scale (Table 3). Of note, after completing the intervention, participants recognized the severity of clinician stigma toward PSU; they recognized the intervention is needed to better understand and demonstrate compassion for mothers and infants affected by PSU.

**Table 2:** Acceptability of the intervention among all participants using the Abbreviated Acceptability Rating Profile (N=25).

Item	Score^a^, mean (SD)
This is an acceptable program for addressing clinician stigma towards perinatal substance use.	5.20 (0.57)
This program should be effective in reducing clinician stigma towards perinatal substance use.	5.04 (0.79)
Clinician stigma toward perinatal substance use is severe enough to justify this program.	5.54 (0.64)
I would recommend this program to others.	5.00 (1.00)
This program would not have bad side effects on the clinician participant.	5.50 (0.65)
I liked this program.	5.00 (1.00)
This program is a good way to handle clinician stigma toward perinatal substance use.	5.08 (0.74)
Overall, the program would help clinicians.	5.24 (0.59)

^a^Items were rated on a 1- to 6-point agreement scale, with higher scores indicating greater levels of acceptability.

**Table 3:** Nurses’ satisfaction with the intervention (n=21).

Item	Score^a^, mean (SD)
The overall program was beneficial to me.	5.14 (0.56)
After completing the program, I have a better understanding of compassion for mothers and infants affected by perinatal substance use.	5.29 (0.63)
After completing the program, I have a better understanding of maternal agency related to caring for mothers and infants affected by perinatal substance use.	5.14 (0.64)
Because of this program, I will be more compassionate to mothers and infants affected by perinatal substance use.	5.25 (0.54)
This program was unique, unlike other training about stigma toward perinatal substance use.	5.19 (0.66)

^a^Items were rated on a 1- to 6-point agreement scale, with higher scores indicating greater satisfaction.

### Qualitative Measures

#### Interviews and Themes

A total of 1797 interview segments were coded from the theatre testing sessions. A total of 4 major themes and 16 subthemes were identified. Major themes included appearance, navigation, characterization, and overall platform. A summary of themes is contained in Figure 4.

#### Appearance

The first major theme was related to the appearance of the platform and different aspects of the visual user experience and design. We identified five subthemes of appearance: (1) highlighting, (2) buttons, (3) graphics, (4) icons, and (5) color. Participants reflected on how highlighting was used in the navigation bar to indicate progression, module completion, and photograph selection during the Perspective Taking module. They observed that highlighted buttons helped them with navigation. They also noted that highlighting in the navigation bar provided a clear indication of their progression and appreciated the use of visual elements to denote locked or unlocked modules, noting the strength of the visual design of the platform. Participants frequently compared it with other training modules where highlighting as a visual cue was not present, which led to a lack of motivation by the user to complete a different training.

…the next button is literally highlighted when you’re next and it literally walks you right through it and I think that’s super important for courses like this because sometimes it’s not easy, people are going to give up and just get right through it and not even complete it as they’re supposed to.

Participant 107

Participants also appreciated that highlighting related to locked photos was used during the Perspective Taking module to help them know where to go next.

Participants noted the use of buttons throughout the platform and how they served as visual cues for navigation. They noticed that illuminating the buttons and giving them specific colors provided the signal to move to the next step.

I liked the visual reminders and cues, having the little padlock sign that’s not where you are now. Good use of color and contrast for the buttons to say next or completed.

Participant 106

The “Next” button also encouraged them not to give up before completing the course. They also mentioned that the modal window containing a blue button with a check mark helped them to know that the course had concluded and that they could download a certificate of completion. Overall, the buttons served as visual cues for the participants, and the colors made them easy to understand.

Participants observed the strengths of how the text was formatted and sized, the arrangement of content, the use of animated characters, and photography. While most participants noted specific strengths related to monochromatic colors for all graphical elements, some participants pointed out that this seemed like too much of one color, making it more difficult to find specific elements.

I would maybe emphasize the woman as a different color. It's like the numbers are that color, the Continue button is that color and the woman is that color. I feel like it's a little overload on that color.

Participant 119

One suggestion they made was that the characters in the Key Takeaways module could be easier to understand if they were in full color. In addition, participants commented that the colorization of the graphics helped them understand what was coming next.

Most participants found the typography and the design of iconography and color made it easier to navigate overall. However, some noted that the overwhelming use of blue icons, buttons, and graphics made it difficult to see where to click next. Participants also indicated that icons in the menu choices helped to prompt them on what they needed to complete. They commented that the checkmarks (once completed) that appeared next to each clinical example in the Key Takeaways module helped clarify what needed to be reviewed before they could continue.

The same screen as before, just the first one has a checkbox now, so I would assume to continue through until they all get a checkbox. And currently the finish is grayed out, so I assume I still have something to do on the screen before I can move on.

Participant 111

Participants also noted that check mark icons in the menu choices helped guide them on what was completed and what still needed to be reviewed. The participants generally found the icons helpful and appealing, especially when combined with bolding and bulleted list formatting.

I think definitely the usability of this was a lot more simpler. I feel like other courses that I’ve taken, it's a little bit outdated and it’s a little bit harder to navigate. I like big fonts and all this stuff. It’s just nice. It’s like bubbles and push, click, next, get started. That was really nice.

Participant 103

Finally, some participants observed that using the large checkmark icon at the end of the training was unclear, noting that they were unsure whether to save the certificate of completion or view it. However, they also mentioned that since the only choice was to click on the page, it led them to download the certificate, which clarified the user experience.

Overall, participants commented that the lavender color was a calming element in the platform. They also found the white background and blue colors appealing. Participants mentioned that the color scheme helped them to navigate, pointing out that the grayed-out objects that appeared not to be clickable helped to indicate how the platform progressed. They also noted their satisfaction with how the colored buttons guided participants to click through the different parts of each module.

I think the content is pretty calming, giving the lavender colors and just the very light design in general. There isn’t too much going on, on the page, so it’s not very distractive, and I could see where my tasks are.

Participant 102

Although some participants stated that there was too much blue and purple, others felt that a similar color was helpful for consistent navigation. In general, participants remarked that the consistent blue color for all the buttons made it clear where to click next and helped them know how to navigate.

#### Navigation

The second major theme was related to the user experience surrounding the platform’s navigation and how users were able to interpret navigational elements. We identified three subthemes of navigation: (1) confusing, (2) anticipate, and (3) easy.

Overall, participants remarked that the platform was not confusing. However, some participants commented that tutorial features were overly prescriptive, which confused them about whether they were still in the tutorial or were supposed to begin their reflection in the Perspective Taking module.

I will say there were times I wasn’t exactly sure if I had completed something, or I guess I should say some things were somewhat repetitive, like when I click the picture and then it pops up again and then it goes away, but then I have to click it again. That was kind of the only thing.

Participant 117

Participants reported that they knew what to do after each step and that the platform’s visual cues helped them anticipate the next step. Overall, they observed that the step-by-step process, including check marks to indicate what was completed, the “Next” buttons that were illuminated when they were activated, and the grayed-out imagery to indicate what could not be selected, helped to guide them on what they needed to do next.

Think it was pretty easy to navigate, because once you complete something, it shows you that you’ve completed it. What else you need to do, there’s buttons that says you need to do that. And once you complete a task, the next button or the finish button illuminates, then I feel that’s your clue that you could move on.

Participant 101

Participants described the visual cues as being very helpful in making navigation easy. They also observed that the signup and log-in features were simple and easy to navigate.

…literally for each section, it would explain what the section is, and then in order to continue, you would have to either fill something out, or click the highlighted button.

Participant 102

#### Characterization

The third major theme was related to how participants characterized the platform across engagement, clarity, familiarity, and difficulty. We identified six subthemes of characterization: (1) hard; (2) engaging; (3) overwhelming; (4) pleasing; (5) clear, simple, and intuitive; and (6) familiar.

There were very few instances of participants indicating difficulty, except for the requirement that they would need to respond to questions about substance use when they might not remember a case related to substance use in their caseload. Participants also noted that if they had personal experience with substance use, it would be more difficult to actively participate.

I also don’t have any personal or family relationships with people with substance use. So I think in that sense, I don’t... I wouldn’t have as much hesitation or maybe nervousness in going through this module versus somebody who does have a personal experience. It might be a little challenging, perhaps.

Participant 105

Participants noted that one of the most engaging aspects of the platform was knowing in advance how many scenarios there would be during the Perspective Taking module and not being able to skip around in the platform.

I really liked the format of this. I’ve done quite a few modules in the last few months, and I wish they all looked like this because I think sometimes the ones that are just you’re going through an automatic voiced PowerPoint and then you have a knowledge check every so often can just really drag you down. Anything that allows for more thoughtful learning I think is good. Yeah, I liked it a lot.

Participant 108

They were also very receptive to the active learning format that allowed them to practice a skill instead of reviewing didactic material. In addition, they found the activities on the platform incredibly engaging since they related to a topic they were already familiar with.

I think a lot of them are rather mundane and bleak and you just feel like, oh, I have to do another module. But this one was just intriguing. I think a lot of that was because of the uniqueness of it. So it didn’t make me feel like, oh, geez, another module I have to do. It kept my interest.

Participant 121

A common observation among participants was that the monochromatic color scheme sometimes felt overwhelming to process. However, they reflected that this was modulated by the different photographs used. When presented with the Perspective Taking module, some participants also noted that the task of writing a narrative in 5 minutes felt like a significant task that may not be achievable. Finally, some participants shared that the number of “Next” and “Continue” buttons during the tutorials seemed overwhelming.

In the tutorial, a lot set up the same. I do feel like this one, there was a lot of “Hit the ‘Next’ button,” and then “Hit the ‘Continue’ button,” whereas if you just hit the “Next” button, could you not... Could you do without some of the “Next,” and then “Next,” again, button. It seemed like you had to hit the “Next” button at that end a lot. And I was like, I feel like that’s redundant. If you hit the “Next” button, you should just be done.

Participant 112

There was high satisfaction with the platform layout, presentation clarity, and information flow, and the participants noted that the use of material design principles caused the platform to be visually pleasant.

I think the content is pretty calming, giving the lavender colors and just the very light design in general. There isn’t too much going on the page, so it’s not very distractive, and I could see where my tasks are.

Participant 101

Participants also noted that instructions and guidance were unambiguous and made progressing through the different modules easy. They were especially satisfied with how the design allowed them to see the difference between the writing activities in the Perspective Taking module and the didactic material in the Key Takeaways module. There was consensus on the dashboard’s clarity about how to progress through the platform, and participants found the signup and sign-in flow to be very intuitive and accessible.

There’s four distinct sections. They kind of go in an order. Once you finish one, it highlights that you're supposed to move on to the next. So there's not really any way to get kind of lost.

Participant 105

Participants noted that the Key Takeaways module structure that used cards, modal windows, and short video segments was clearly organized and easy to follow.

Finally, participants consistently reported that the format was very familiar and similar to other courses they had taken.

It reminds me of kind of the courses that we have to take in the hospital when we have to renew or look at our continuing education. It has kind of that similar feel.

Participant 115

#### Overall Platform

The fourth major theme was related to participant observations about the entire platform experience, with a particular focus on their suggestions for improvements and how they would characterize the time commitment required to complete the platform activities. We identified two subthemes: (1) suggestions and (2) time-consuming.

Participants suggested that during sign-up and profile creation, the platform should not request a profile picture, as this is too personal for an educational module connected to their workplace.

The profile picture thing, unless it was like pre-selected profile pictures, but I think it would just be kind of funny also if you were just doing an education module for them to ask for a profile picture.

Participant 116

In the Pre-Course Self-Assessment module, participants noted that it would be helpful if they were given more notice that they would be prompted to share their personal perspective as part of the training, which may be something not all nurses want to do. During the Perspective Taking module, participant suggestions included reducing the length of the tutorial to avoid redundancy, making the visuals more explicit about what role they were assuming in each of the narrative writing steps, retaining instructions from one screen to the next to help recall what to do, and adding more arrows and icons to help with users who are less experienced with web-based platforms. In addition, participants suggested some improvements to the exercise that was being facilitated asynchronously in this module. This included a desire to learn other participants’ perspectives and eliminate the review of their narratives after they wrote them, since they could not be edited. In the Key Takeaways module, participants suggested that the clinical examples and strategies could be separated more clearly to deliver more independent information. They also thought that these could be more consolidated to help participants remember them.

It might be nice if you were like, here, read this clinical example and then click a button and you say, actually, here’s the strategies that we recommend for this situation, instead of just having it all on one page. If you separate those, it might be more clear to people this situation actually did not go well. Here's what we want to promote.

Participant 106

Finally, some participants suggested that the platform be more explicit about available subtitles for videos so that users who do not have headphones could still review the video content. In the Course Completion and Certificate module, participants suggested that the platform include an instrument that assesses competency related to the learning before providing a certificate, as well as a presentation of statistics and background data about maternal substance use, so that participants could carry more issue-related content with them after participating.

Very few participants noted that the platform was too time-intensive after completing the application session during the interview. Those participants who did observe this aspect said that when using the platform, they might need to take a break after the perspective-taking exercise before proceeding to the Key Takeaways module, since the perspective-taking exercise required writing, and the Key Takeaways module required listening and reading. Even though the total amount of time for the whole session was approximately 30-45 minutes (interview and application session together), they thought that taking a break between the two modules might be helpful.

...I see a few key takeaways, but by this point I’d probably take a two-minute break or five-minute break only because that perspective taking took a lot of my mental capacity, so I’ll probably just rest and, I don’t know, refresh my mind for a couple minutes. Then, I’ll go ahead and press the key takeaway.

Participant 103

## Discussion

### Principal Findings

In this study, we evaluated the initial usability, acceptability, and feasibility of the interactive, responsive platform version of ArtSpective for PSU—an adapted version of an arts-based synchronous intervention designed to improve nurses’ stigmatizing attitudes toward PSU—following a mixed methods approach. We engaged 21 perinatal nurses and 4 experts in instructional design, stigma, and implicit bias to participate in theatre-testing sessions where they qualitatively assessed the usability of the platform and quantitatively assessed acceptability. This mixed methods approach has been extensively used to evaluate usability [61-64] and is consistent with previous work that uses theatre testing to gather feedback on an intervention from a specific group of users [65-67].

The results from the theatre testing sessions were positive overall, with participants expressing high satisfaction with the appearance of the platform, the ease with which they were able to navigate the various modules, their engagement in the platform, the clarity of the presentation, and its feasibility to be completed asynchronously by perinatal nurses. These findings were consistent with the quantitative acceptability measures and complementary satisfaction items. Using a mixed methods approach provided a more holistic assessment of the platform’s different usability, acceptability, and feasibility aspects among perinatal nurses and experts.

Furthermore, while quantitative and qualitative analyses indicated high acceptability and satisfaction, the theatre-testing sessions allowed for more granular data on platform usability. The use of theatre testing as a modality for conducting this type of evaluation also provided a more accessible method for practicing nurses to participate as it did not require them to allocate more significant time to engage in task-oriented usability testing, overcoming barriers related to engaging health care providers in this type of study [68-70] and engaging far more than the 5 participants that are minimally adequate to identifying more basic usability challenges [71]. Furthermore, an important implication of using theatre testing in this way is that we could collect granular usability data without investing fully in a developed platform. This provided for a more cost-effective development process that allowed developers to incorporate target user feedback before the final development of the platform.

**Figure 5:**
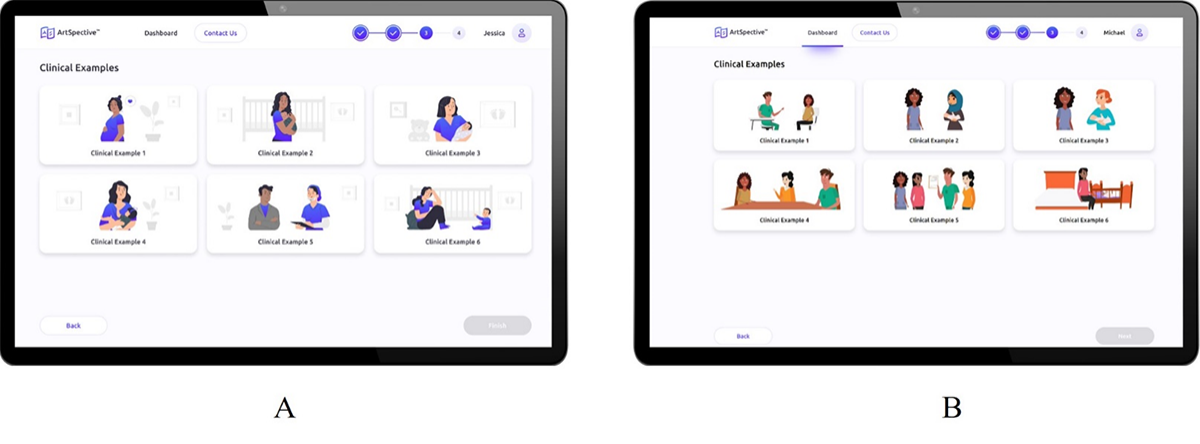
Platform screens before and after the completion of theatre-testing sessions: (A) the original Clinical Examples screen with monochromatic characters and (B) the updated screen with full-color characters aligned with the animated videos.

In designing an interactive, responsive platform for perinatal nurses, we observed that while having a monochromatic color scheme consistent with material design principles may be helpful, it might also make it more difficult for nurses to discern differences. As a result, one of the updates made to the platform before development included changing the color scheme of illustrated characters in the Key Takeaways module from monochromatic to full color (Figure 5). In addition, while evidence related to effective asynchronous module instructional design recommends robust tutorials to guide participants [72,73], we observed that too many instructions could lead to more confusion and frustration. Therefore, future tutorial integration into interactive, responsive platforms for health care providers should be highly targeted and aligned with only the most essential information. This could be integrated through tooltips or info buttons that allow users to select which aspects of the platform they need more help clarifying. At the same time, our findings demonstrate that while an asynchronous platform may facilitate engagement in an intervention among health care providers, it must facilitate rapid content completion without sacrificing meaningful comprehension and overall effectiveness. Our findings indicate that achieving this goal requires a dynamic and modern design that also balances graphical and verbal cues with a similar structure to other training platforms that allows users to recognize that the material is consistent with different pieces of training and, therefore, gain trust with users so that they are willing to engage in completing the intervention with a high degree of fidelity to each step.

### Comparison With Previous Work

Our findings related to acceptability and satisfaction were consistent with those related to our previous work, demonstrating satisfaction with the intervention [24]. This supports our conclusion that the platform addressed some of the dissatisfaction points identified in the previous study of the synchronous intervention, which illuminated the need for an asynchronous version of the intervention that would not compromise satisfaction overall among nurses.

### Limitations

Our study is not without limitations. First, while we achieved information power after recruiting 21 perinatal nurses, 28% (7/25) of participants identified as either African American or Asian, and 88% (22/25) identified as female. While these demographics are somewhat representative of the perinatal nursing field, our results related to the usability of an interactive, responsive platform outside of the perinatal specialty might be more generalizable with a different group of participants. Furthermore, all participants were from two states in the Midwest, which may not be generalizable to providers in other regions of the United States. Second, although a larger sample of experts may have offered additional feedback, the sample included in this study was conveniently selected, represented multiple disciplines with varied expertise, and the research team concluded that information power was reached after receiving very similar feedback from all 4 experts being interviewed. Third, while theatre testing was an effective approach to gathering input from target users, we could not incorporate task-specific analyses into the sessions, as the theatre-testing approach is oriented around the presentation of the prototype. This enabled our study to include more nurses, but a task-oriented analysis, where we could compute a severity score for different issues encountered, may have allowed us to further augment our findings [74-76]. Fourth, while the validity and reliability of the AARP scale have been previously demonstrated [56], we did not have a sample large enough to evaluate the reliability of the AARP or the individual satisfaction items in our study. However, these items were administered primarily to pragmatically assess acceptability and satisfaction overall to inform future usability testing of the developed platform. Finally, since we were presenting a prototype of the intervention, not all features were fully implemented. For example, users reviewed a desktop version of the platform even though it was designed responsively. The decision to only review the desktop version was motivated by study feasibility. A prototype that switches between desktop and mobile formats may have confused participants as they went through the various modules. One alternative would be to review a separate mobile prototype. However, that would introduce feasibility challenges related to how much time perinatal nurses could allocate to participate in the study. To overcome these limitations, future studies might stratify participants into two groups where one group engages in theatre testing with a desktop-oriented prototype and the second group reviews a mobile prototype.

### Conclusions

Developing and evaluating the usability of a digital adaptation of ArtSpective for PSU provided insight into key aspects related to usability, acceptability, and feasibility that should be considered when creating a digital adaptation of an arts-based intervention for health care providers. Overall, theatre-testing sessions identified key strengths and weaknesses of the prototype and provided opportunities to refine the platform’s design before engaging in more costly development. Nurses and experts found the platform acceptable and were highly satisfied with its design and features. Future studies could evaluate the efficacy of the fully developed platform among practicing perinatal nurses and inform further refinements of the platform to ensure that health care providers in other specialties can engage with the intervention asynchronously.

## Appendix

Multimedia Appendix 1 Semi-Structured Interview Guide.

Multimedia Appendix 2 COREQ (COnsolidated criteria for REporting Qualitative research) Checklist.

## Abbreviations

AARP: Abbreviated Acceptability Rating Profile
ADAPT-ITT: Assessment Decision Administration Production Topical Experts-Integration Training Testing
COREQ: Consolidated Criteria for Reporting Qualitative Research
PSU: perinatal substance use
