# Supplementary material for: Evaluating the Usability, Acceptability, User Experience, and Design of an Interactive Responsive Platform to Improve Perinatal Nurses’ Stigmatizing Attitudes Toward Substance Use in Pregnancy: Mixed Methods Study
Source: JMIR Hum Factors. 2025 May 8;12:e67685. doi: 10.2196/67685 (PMC12099274; doi:10.2196/67685)
Supplement: Multimedia Appendix 1 [file humanfactors_v12i1e67685_app1.docx]

**ArtSpective Semi-Structured Interview Guide**

**Intro script**

Hello ____, my name is ____ and I am part of the ArtSpective research team. Thank you for your participation in this interview. We are interested in your thoughts about the ArtSpective Program and your participation in the study. We anticipate this interview will take around 20 minutes but will not go over 30 minutes. As a reminder, this interview will be audio recorded so that we don’t miss anything you have to say. When you enrolled, you were provided with information about the study and how your identity and data would be kept confidential. There are no right or wrong answers. Do you have any questions for me about this?

Is it okay if I start the recording now?

Okay, I am now recording. *{Start recording.}*

| Questions | Probing questions |
| --- | --- |
| In July or August, you completed the ArtSpective program. Tell me what you remember about the program.  What was your overall impression of the ArtSpective Program?  What aspects of the ArtSpective Program did you find the most interesting or engaging? What aspects were most helpful to you as a nurse?  What aspects did you not find interesting or engaging? What would you change?  Did you find the activity component appealing? How did this differ from other trainings you’ve taken as a nurse?  How did the interactive components of the program motivate you to complete the training?  Would you do a program like this again? Why or why not?  If your hospital mandated this training, would it change the way you engaged with it?  What challenges or obstacles, if any, did you encounter while completing the ArtSpective Program? | [if they cannot remember]: The ArtSpective Program involved a perspective taking exercise that had you pick 2 photographs and write stories about them – 1 story from the nurse perspective and 1 story from the perspective of the individual in the photo. Then you watched animated videos about the importance of recognizing that your perspective of others may not be accurate and could be biased. Finally, you reviewed clinical stories and related strategies to reduce misperceptions of mothers with substance use disorder.  Were there any particular aspects of the content or structure that stood out to you?    Were there any interactive elements that you found engaging? Not engaging?    Were there technical issues, time constraints, or content difficulties that affected your progress?  Were there any specific transitions that you found confusing or unclear? |
| Let’s talk about your role as a research participant in this study.  How did you hear about the study?  What motivated you to join the study as a participant?  Did you experience any challenges enrolling in the study? How about transitioning between the 3 phases? How do you think these challenges can be addressed in the future? | Did you receive an email from the study team? Your nursing leader?  How do you think these challenges can be addressed in the future?  How could the process be streamlined to make it easier for participants to complete all phases? |
| What changes or improvements would you recommend to make the ArtSpective Program for future participants?  What changes or improvements would you recommend to make participating in the study better for participants? |  |

**Qualtrics Survey script**

We almost done. I am sharing a link in the chat for a short survey – the same one you completed in Phase 2. Please click the link and complete the survey. After you’ve finished the survey, please let me know, as I have one last question to ask you before we conclude.

[SHARE SURVEY LINK IN CHAT]

| Question | Probing questions |
| --- | --- |
| After completing the ArtSpective Program, did you use any aspect of the training in your clinical work? In your life outside of work?  Do you think the ArtSpective Program can help others identify the perspectives of others and reduce implicit bias and stigma towards substance use in pregnancy? Why or why not?  Is there anything else you’d like to say about the ArtSpective Program or the research study? | Did you think about perspective taking when interacting with patients? With people in your community? |

This concludes our interview. Thank you for your time and feedback. Incentives will be mailed to you soon. Have a great day!
